# Migrant-sensitive healthcare in Europe: advancing health equity through accessibility, acceptability, quality, and trust

**DOI:** 10.1016/j.lanepe.2023.100805

**Published:** 2024-05-28

**Authors:** Sibel T. Savas, Michael Knipper, Diane Duclos, Esther Sharma, María Idoia Ugarte-Gurrutxaga, Karl Blanchet

**Affiliations:** a Institute of the History, Theory and Ethics of Medicine, University Justus Liebig Giessen, Leihgesterner Weg 52, Giessen 35392, Germany; b London School of Hygiene and Tropical Medicine, 5-17 Tavistock Place, London WC1H 9SH, UK; c London School of Hygiene and Tropical Medicine, Keppel Street, London WC1E 7HT, UK; d Department of Nursing, Physiotherapy and Occupational Therapy, University of Castilla-La Mancha, Toledo Campus, Avda Carlos III, Toledo 45071, Spain; e Faculty of Medicine, Geneva Centre of Humanitarian Studies, University of Geneva, Boulevard du Pont-d'Arve 28, Geneva 1205, Switzerland

**Keywords:** Migration and health, Cultural competency, Cultural sensitivity, Health and human rights, Quality of care, Universal Health Coverage

## Abstract

The advancement of migrant-sensitive health care in Europe has been a topic of many initiatives and academics debates for over 20 years in Europe, yet with rather limited progress in terms of comprehensive and sustainable implementation. We argue that a human rights-based approach with clearly defined duties and responsibilities of governments, relevant public and private institutions as well as professionals is needed, in line with a sound understanding and thoughtful implementation and further development of concepts and standards for providing migrant sensitive care as an essential component of Universal Health Coverage. We suggest drawing particular attention to the interrelated features of accessibility, acceptability, quality, and trust to inform policies and practice. Innovative approaches with substantial involvement of social and cultural sciences are needed for adapting clinical care and health services to the growing social and cultural diversity of European societies.

**Key messages**

1. Little progress has been made towards a comprehensive implementation of migrant and culturally sensitive healthcare in Europe over the last 20 years, despite significant advancements on conceptual and operational levels.
2. The current social and political climate in Europe, with deterring migration policies and poor support for migrant-sensitive care in many countries, puts a strain on healthcare and other professionals committed to human rights and health equity and undermines Universal Health Coverage.
3. According to universal human rights, all human beings are “right holders”, independent of migration background or legal status, and thus entitled to scientifically–and medically–appropriate care without discrimination.
4. In order to advance migrant-sensitive care, the respective duties and responsibilities of states, governments, institutions and individuals need to be defined, according to international law and professional ethics.
5. Accessibility, acceptability, and quality of care, as well as trust in health services and professionals, are critical domains for informing migrant-sensitive health services and care.
6. A theoretically sound understanding of culture that fosters meaningful engagement with individuals and communities and attention to the real living conditions, experiences, and concerns of the people, is crucial for migrant-sensitive health services.
7. Innovative approaches with substantial involvement of social and cultural are needed for adapting clinical care and health services to the growing social and cultural diversity of European societies.

## Introduction

Adapting European health services to migration and cultural diversity is yet to be accomplished. Despite multiple initiatives, proposals, “calls for action” and programs for the last two decades, health systems and services in Europe are still far from adequately responding to the diverse cultural reality of contemporary European societies. For refugees and migrants, it is still a matter of chance whether they will enjoy people-centred health care that adequately responds to their individual needs. Health services in Europe are not free of xenophobia, racism, and discrimination in its multiple overt and covert, interpersonal and structural forms. We are still far from having migrant- and diversity-sensitive health care “integrated in the mainstream of health services, embedded in policy and supported by communities”, as David Ingleby called for in a critical review in 2011.^1^ As a result, social, cultural, structural, language and legal barriers keep preventing many migrants (including undocumented migrants), refugees, and displaced groups from accessing health services in the same reliability and quality as compared to host populations.

Little progress has been achieved even amongst the most wealthy European countries, despite strong political commitments,^2^ and numerous initiatives showing the way forward.^3^, ^4^, ^5^, ^6^, ^7^, ^8^, ^9^, ^10^, ^11^, ^12^, ^13^ On the other hand, all these contributions have yielded great progress in terms of theory and methodologies for implementation: We now understand notions like culture- and migrant-sensitive health services much better, and how these concepts can be translated into practice. Controversies regarding—for example—suitable definitions of “culture” or the relevance of “ethnicity” in medicine and health, have advanced our knowledge on *positive* ways for using these terms for improving the health and dignity of patients and communities through better communication, understanding, and care.^14^, ^15^, ^16^, ^17^, ^18^ At the same time, we have become better aware of the *negative* effects of inconsiderate and theoretically-uninformed use of these terms leading to stereotyping or sustaining overt or covert forms of racial discrimination.^19^^,^^20^

But there are further reasons that make talking about migration, culture and health equity in Europe today in 2023 different from twenty years ago, when the broader discussions on migration, integration and health in European countries had just started.^21^ The painful reality of close to 28.000 migrants fatalities documented in the Mediterranean since 2014,^22^ of illegal pushbacks at European borders by government authorities, as well as the surge of toxic “narratives of hate”^23^ regarding migrants, were probably unimaginable twenty years ago. Moreover, the experiences of austerity measures in Europe disproportionately affecting health care for migrants during the European Financial Crisis,^7^^,^^24^ the European reception crisis of the years 2015–2016, the COVID-19 pandemic and, recently, the massive displacements following Russia’s invasion of Ukraine need to inform our view on migration, culture, and equity in healthcare today.

Against this background, this article offers a critical view on the attempts, advancements, and challenges for providing inclusive, migrant-sensitive health care in Europe. Based on a purposeful review of academic literature from multiple disciplines, such as medical anthropology, public health, medical education, and humanitarian medicine, we aim to provide a critical synthesis of the main contributions, experiences, and persisting or newly defined challenges. The overall framework to guide our analysis was defined by the ethical commitment to health equity as well as the legal and conceptual components of the Right to Health,^25^ in correspondence with a “critical perspective on migrant health”.^26^

**Search strategy and selection criteria**

References for this Personal View were identified through searches of PubMed with the search terms “culture”, “migra∗”, and “healthcare”, “migra∗” and “right to health”, as well as “migra∗”, “healthcare” and “trust”, published between 2010 and August 2023. Articles were also identified through Cited Reference Searching related to pivotal publications issued since 2000, and searches of the authors’ own files and collections. Papers, monographs, collected volumes and reports published in English, German and Spanish were considered. The final reference list was generated on the basis of originality and relevance to the aim and the overall framework of this Personal View.

## Migration, diversity, and the right to health

According to the 2016 “Strategy and action plan for refugee and migrant health” of the World Health Organisation (WHO) Regional Office for Europe, “access to responsive, people-centred health systems is essential to ensure available health care for all refugees, asylum seekers and migrants throughout the migration journey”.^2^^:9^ The WHO document then draws attention on the multiple “formal and informal barriers to health care” for refugees and migrants that can be subsumed under three major categories: structural, social and cultural factors.

For assessing and overcoming the well-known barriers to equitable health care for all refugees and migrants in Europe, we suggest to adhere to the four “core elements” of the Right to Health as defined by the UN Council on Social, Economic and Cultural Rights in 2000^25^: the “availability, accessibility, acceptability and quality” of health facilities and services for all. However, given that the physical availability of health services is largely ensured in Europe, we will concentrate on accessibility, acceptability, and quality of health services, but add a further dimension of cross-cutting relevance: trust.

Human rights do not materialise by themselves. Advancing human rights implies investment of time, energy, empathy, and other resources (not the least financial) especially for those people who are at the highest risk of suffering marginalisation, neglect, discrimination, and exploitation in a given society. A fundamental tenet of human rights is therefore the differentiation between right holders and duty bearers: All human beings—including all refugees and migrants—are “right holders”, with human rights and dignity not being conditioned to any kind of merit or related to any obligation. The responsibility for protecting, respecting, and fulfilling human rights lies, in contrast, with the States: States are “duty bearers”, and the current observation of European countries systematically disregarding universal rights of migrants only reaffirms the need to hold governments accountable.^27^ However, the realisation of human rights should not be left to States and international organisations like the WHO: civil society plays an essential role by supporting, watching, and forcing States in multiple ways to comply with human rights.^28^ This is particularly relevant for the health sector: rights-based approaches provide health professionals with opportunities to promote health equity that go far beyond legal mechanisms and obligations.^29^, ^30^, ^31^ In addition to take states and governments as “duty bearers”, we thus suggest to understand professional actors in all branches of the health system as “responsibility holders”^30^ for better defining tasks and preventing the dilution of responsibilities.

## Accessibility, acceptability, quality, and trust

Accessibility, acceptability and quality of health services and care, as well as trust into services and health professionals, are critical criteria to be met for truly complying with the universal right to health in Europe. The differentiation of the four domains helps to systematically identify challenges and solutions that, in line with a clear assessment of duties and responsibilities, may lead to concrete actions.

### Accessibility

In contrast to many regions of the world, the physical availability of health services in Europe is largely achieved. However, health services are often not accessible for refugees and migrants, due to economic, legal and many other reasons. Barriers are particularly high for undocumented migrants, asylum seekers and refugees, often conditioned by the multiplicity of legal categories applied to people seeking protection.^32^

As health systems of European countries are extremely diverse and health and migration policies are in constant change, the situation is different in every country, with large regional variances within some European countries, making the situation even more complicated.^12^^,^^13^ An exception of the rule is the inclusion of Ukrainian people fleeing the war after Russia’s invasion of Ukraine through the EU Temporary Protection Directive issued on March 4, 2022. In terms of health equity and universal human rights, this political decision by the European Union is both encouraging and deeply disappointing: It shows that inclusive health policies are possible and can be implemented in case of an emergency at very short notice. Yet, the distinction between Ukrainian nationals and other people fleeing the war in Ukraine, as well as the chasm regarding the treatment of refugees and displaced people from all other regions and conflict zones like Syria, Iran, and Afghanistan, is in clear opposition to universal human rights.

In addition to this political and legal dimension, health systems must be structurally prepared and equipped for receiving refugees and migrants. Health systems need support, both economically as well as through guidance, training and task-sharing between regular health services and humanitarian organisations.^33^ A further aspect of accessibility is access to information: migrants, refugees and displaced people need adequate information about entitlements and how to navigate the health system, considering their language and their actual living conditions.

### Acceptability

According to the UN General Comment on the Right to Health (paragraph 12),^25^ this domain points to the need that “all health facilities, goods and services must be respectful of medical ethics and culturally appropriate”, giving explicit mention to “individuals, minorities, peoples and communities”, as well as sensitivity to “gender and life-cycle requirements”, and “confidentiality”. By highlighting medical ethics, it establishes a direct link to health professionals as “responsibility holders”, who are primarily in charge of fulfilling acceptability. However, even the most-well intentioned and best trained professional will be unable to provide emphatic person-centred care under conditions of limited time and resources, without encouragement and support within the team and the overall institution. Moreover, reliable support by interpreters, social workers and professionals of other areas is needed to attend to the intersecting needs of people in situations of migration or displacement. Providing the structural conditions for acceptability of health services corresponds to the “duty bearers”: governments and public institutions in charge of administrating health services.

A central feature of acceptability is the need to provide culturally sensitive care, and we have seen massive strides towards a better understanding of how to embrace culture and how to deal with cultural diversity in health services and care.^4^, ^5^, ^6^, ^7^, ^8^, ^9^, ^10^, ^11^, ^12^, ^13^, ^14^^,^^26^^,^^34^^,^^35^ However, broad implementation is still lacking both at the level of training and medical education, as well as in the daily routine of health services and humanitarian assistance. Culture and cultural diversity in the context of medicine and health are still too often addressed through stereotype assumptions regarding the “culture” of specific groups. From a health provider’s perspective, this may be perceived as useful and practical, as it provides apparently rapid answers to complex questions. But this approach ignores the cultural heterogeneity within the assumed groups and neglects the dynamics of change and cultural development over time, as well as the individual patients’ real-life experiences, perceptions, and problems. Instead of advancing person-centred care, the stereotype use of “culture” is prone to confound the individual person or community with a cultural (and sometimes racist) cliché. This is particularly relevant for people living for years in unstable legal and social situations, in camps or transit, defined by Kafkaesque legal provisions and without being able to settle down and build a future in safety and dignity. Considering “culture” should thus only be the starting point for approaching a patients’ situation and perspective, addressing all relevant social and structural aspects.^14^^,^^18^^,^^36^

Another essential aspect of culturally-sensitive care is to include critical self-reflection and awareness, as expressed for example with the concept of “cultural humility”.^31^^,^^37^ The “clinical gaze”^38^ of physicians and the institutional culture of health facilities shape how health professionals perceive and treat patients, and how their particular experiences, needs and concerns are given credit within daily routines.^16^ Power-relations during clinical encounters need to be reflected for fostering non-paternalistic attitudes, as well as the critical question for the legacies of colonialism in our minds and attitudes: The historical roots and hidden structures of racism and exclusion—both in the global health architecture and in our European societies—need to be addressed seriously by health professionals and medical institutions.^39^, ^40^, ^41^

### Quality

Regarding the fourth essential element of the Right to Health, the UN General Comment states that health services must be “scientifically and medically appropriate and of good quality”. This rather general statement leaves much room for discussions and controversies, but there are at least three aspects of overwhelming importance: the unacceptability of “double standards” with providing health care of lower quality to certain people and groups, the need for adequate communication across language barriers, and continuity of care in case of chronic diseases or conditions with the need for long term treatment.

Double-standards in terms of sub-standard care for specific people of population groups according to migration status, nationality or on any other grounds is a direct infringement of the principle of “universality” of human dignity and rights. In case of emergency situations with massive population movements, a focus of health care delivery on emergency care or acute medical issues might be justified as an initial response, but transformation into a comprehensive approach needs to be envisaged right from the beginning and implemented as soon as possible.^33^ The legal or structural institutionalisation of double-standards, for example through legal provisions that define reduced entitlements for asylum seekers or undocumented migrants, is unacceptable from a medical, ethical and human rights perspective. Moreover, policies and laws of this kind put health professionals in difficult situations by forcing them to deliver care that contradicts professional ethical standards and universal human rights.

However, double-standards may also emerge on the level of individual care provision, when for example social discourses regarding the “deservingness” of migrants for social services gain influence in the physician-patient relationship.^42^^,^^43^ Considering people as “unwanted, undesirable, and undeserving of protection or public support”^26^^:8^ and categorising them as the "other”,^26^^:6^ prepares the field to accept the provision of sub-standard care at an individual level—even more, when legal frameworks and policies sustain the idea that discrimination is acceptable.

The second aspect of quality to be mentioned here is communication and cultural mediation. High-quality communication both in medical and linguistic terms is an absolutely essential aspect of health services, and this also applies to patients, people and communities who do not have the language skills of the country where they happen to be in need of medical care.^44^^,^^45^ Considering the obvious importance of quality communication for health care provision, it is quite troubling to read in a relevant document published in 2019 by the European Office of WHO, that “the beneficial impact of intercultural mediators is hindered by a lack of professionalisation, insufficient training and the non-systematic and inconsistent implementation of intercultural mediation programmes.”^44^, ^45^, ^46^, ^47^^:ii^ As bad communication is known to severely undermine quality of care and trust,^48^, ^49^, ^50^ both duty bearers and responsibility holders need to comply with their respective commitments and tasks.

The third dimension of quality of care is the need to assure continuity of care, both in relation to the migration journey with continuity of care over all phases of the migration trajectory, but also in a country of prolonged transit (that may last years) and resettlement. Chronic health conditions like non-communicable diseases, as well as mental health problems and infectious diseases with a long-term treatment like TB and HIV/AIDS must not be interrupted due to migration movements or sub-standard provision of medical care.

### Trust

In addition to accessibility, acceptability, and quality of care, we suggest including trust as a further dimension that deserves systematic attention. According to WHO, “trusted health systems” that respond to the “needs and preferences [of patients] in humane and holistic ways” are essential for person-centred care.^51^^:7^ Yet, trust must be earned and maintained, and can easily be undermined and hampered. The recent Covid-19 pandemic has shown again,^52^ that migrants are often among those who have the least trust in the health system of a given country.^48^^,^^49^^,^^52^

The creation and preservation of trust in health systems and professionals depends on multiple interrelated factors, with responsibilities at all levels of a health care system and beyond. At the level of individual care, trust is shaped by the personal attitude of the professionals. Empathy, cultural sensitivity, and quality communication, if needed with the support of cultural mediation and interpreters, are essential for creating trust. On the contrary, health professionals who are perceived to be uncaring and inconsiderate towards migrants' needs will substantially reduce trust in health care, even beyond the individual situation.^50^^,^^53^^,^^54^

A further important factor for trust in health services is located rather outside the health system, in the social, legal and political context: Living as an undocumented migrant, for example, with fear of deportation is marked by insecurity and well-founded skepticism regarding public institutions, undermining also the trust into health services.^32^^,^^55^^,^^56^ Experiences of exclusion, marginalization, racial discrimination and violence during the migration process or in transit and host countries also undermine trust. Even more so, when considering that this might be related to micro-traumata, that are prone to lead to a severe deterioration of the individual’s mental health.^54^

Creating and maintaining trust of migrants, refugees and displaced people in the health system is an important avenue for improving migrant-sensitive health care. All measures promoting acceptability and quality of care are directly related to increasing trust. Moreover, participatory approaches and community engagement are of highest value to create trust, from the individual to the community level.^57^, ^58^, ^59^

## Perspectives for action

Adapting health services and care to the social reality of societies shaped by the multiple types of past, recent and present migration is a comprehensive task that requires sound and sustained action by all individual and institutional actors involved. For practical action, we suggest the following complementary points.

### Fulfilment of duties and responsibilities at all levels

Migrant-sensitive health care can only be achieved when all stakeholders–duty bearers (governments and public agencies) and responsibility holders (professionals in the health sector, including health administration and financing)–fulfil their duties and responsibilities. Unfortunately, the investment of time, empathy, financial and other resources into person-centred health care of people who are at the social margins of our societies, affected by racism, xenophobia and discrimination on other grounds, far too often relies on the personal engagement of individuals and non-government organisations with unstable funding and weak structures. Austerity measures and privatisation of health services have proven to compromise investments in health equity even more. Yet, as a central pillar of Universal Health Coverage (UHC), the fulfilment of comprehensive, person-centred health services for all is in the best interest of all and must be treated as such—in health policies, financing, training and daily health care delivery.

### Innovative approaches for recontextualizing health services and care

In view of the growing social and cultural diversity in Europe, the uncritical use of categories like “ethnicity” and “country of origin” is of little to no value for health research, professional training, and care. A critical understanding of "culture" is needed, with explicit attention to social, structural, legal, and political contexts and determinants of health, as well as the intersection of the different factors driving inequities and discrimination, like gender, class, ethnicity and race.^14^, ^15^, ^16^, ^17^, ^18^^,^^26^^,^^36^^,^^37^

Innovative forms of interdisciplinary collaboration between medical, social and cultural sciences need to be implemented, building on the close ties between medicine, anthropology and history: In clinical practice, the anthropological perspective advances the empathic “curiosity”^45^ of health professionals towards patients and communities for getting closer to what is “really at stake”^14^ for the people. An “expanded social history”^60^ puts an individuals’ medical condition in relation to his or her personal history, enriched by the trained health professional's knowledge on how migration contexts may affect health. Sensitivity to context, living conditions, and lived experiences, as well as collaboration with non-medical partners (like social work, legal counselling)^18^^,^^30^ enables clinicians to promote “health and the capacity to flourish”^31^^:1^ of migrant patients and communities.

On the research side, the multiple determinants of health–including racism and xenophobia–have to be disentangled^61^, ^62^, ^63^ with anthropological studies providing useful knowledge on the lived reality of refugees, migrants and displaced people to inform clinical practice and public health.^26^^,^^38^^,^^56^^,^^64^ The social, cultural and structural dimensions of health services and care need to be addressed and delivered with the same level of professionality and academic commitment as the medical and biological aspects of health and disease. Cultural stereotypes, structural violence, racial discrimination, or poor quality of physician-patient communication are equally harmful for patients as incorrectly dosed pharmaceuticals or badly performed surgical interventions.

### Deployment of existing technical guidance and standards

More than two decades of sustained engagement of multiple researchers, professionals of health and allied sciences, technical experts, and activists have yielded significant results in terms of publications, examples of good practices, consensus documents, handbooks, and guidelines for multiple aspects of migrant-sensitive health services.^8^^,^^10^^,^^11^^,^^16^^,^^17^^,^^35^^,^^44^^,^^65^, ^66^, ^67^ The first step for advancing accessibility, acceptability, quality, and trust in healthcare for refugees and migrants would thus be to make use of the existing standards by thoughtfully applying and adapting recommendations and guidelines to the particular local and professional context, including teaching and training.

## Conclusions

Adapting health services and care to the social reality of societies shaped by the multiple types of past, recent and present migration is a challenging but necessary task to achieve UHC. Migration and the growing social, cultural, and linguistic diversity of our European societies are not a transitory social phenomenon, but a reality that needs to be attended in terms of prosociality: attending “the needs of the society as a whole”.^68^^:1227^ This is not least one painful lesson of the COVID-19-pandemic. Unfortunately, the widespread lack of coherent, constructive, human rights-based migration and health policies puts severe strains on health services, professionals and, above all, the health and dignity of refugees and migrants in Europe.

However, advancing the realisation of the right to health for all members of our society essentially depends on all actors upholding their duties and responsibilities for overcoming the social, structural, and cultural barriers to migrant-sensitive care. The emphasis of this article on accessibility, acceptability, quality, and trust is a proposition and invitation to health professionals and all actors and stakeholders involved—those in close contact to patients just as others engaged at the administrative, economic, or political level—to reflect on their share of personal and professional responsibility and scope of action. The harmful or beneficial outcome of health policies and services are jointly crafted by all parties involved in the complex processes of protecting, achieving, and maintaining health.

**Case studies**

**Culturally sensitive approach to prevent female genital mutilation in Spain**

Female Genital Mutilation (FGM) is a form of gender-based violence and its eradication requires a change of attitude in those who practice and defend it. This can only be achieved when professionals and activists are able to establish a relation of trust and meaningful dialogue with mothers, families, and communities, despite the enormous culture shock that this practice evokes. Empathy, cultural humility, and respect need to be combined with a sustained commitment to universal human rights, including the conviction that not every cultural practice is acceptable.

Doctors of the World Spain proposed the creation of an Interdisciplinary Commission for the prevention of FGM in girls in a village in Toledo (Recas) with a high percentage of population of Malian origin.^30^ The existence of a Regional Protocol for the Prevention of FGM facilitated coordinated efforts, starting in 2019 with convening local professionals from the fields of health, education, and social services. Space was created for raising awareness about the role of each of the professions in the identification and prevention of the risk of FGM in girls, appealing to their co-responsibility from the ethics of care and universal human rights. Training in cultural competence and relevant social factors of the migratory context was provided, highlighting the importance of not judging mothers and/or fathers defending FGM. Rather, the focus was on understanding the social and cultural meaning of this practice for them. Many members of communities practicing FGM are themselves ambivalent regarding this violent practice, aware of the suffering it causes for their daughters or the legal problems it causes in Spain. By a participatory approach, the community was involved in the whole process, with motivational interviewing supporting mothers and/or fathers to finally build their arguments for change.

In the event of identifying a girl at risk for being exposed to FGM, the protocol is activated so that concrete action can be taken. As a result of this interdisciplinary coordination, in 2022 there were six cases of activation of the protocol. Another five were handled directly by health and education centres.

**Trust in health systems and the politics of deterrence in the UK**

For several years, politics of containment and deterrence targeting migrants, including refugees, have been implemented across Europe, reducing access to health services and impacting experiences of care. In the UK, the government has pursued a policy of deliberately creating a ‘hostile environment’ for certain categories of migrants, which includes additional barriers to access national health services involving ID checks, sharing of confidential non-clinical data between NHS Digital and the Home Office and introducing user fees for secondary care.^69^ Legacies of government bodies using information held in primary care records to monitor migrants have instigated persistent fear for certain patients to seek healthcare, including registering with a GP practice. In this context, the erosion of migrant patients’ trust towards the NHS cannot be reduced to issues of inter-individual interactions. For some, accessing healthcare means encountering authorities which have repeatedly failed and systematically discriminated against them, and results in exposing oneself to deportation. Some health professionals have resisted, either by refusing to ask service users about their immigration status or by actively campaigning to bring an end to these charging policies, such as the Patients not Passports campaign.^70^

**Maternal and new-born health care for refugees in Serbia**

Serbia is an established transit country for refugees travelling overland to the EU. The United Nations High Commissioner for Refugees (UNHCR) estimated that there were over 4000 registered refugees in Serbia at the end of 2022, while the number of non-registered refugees living in informal squats is unknown.^71^

Health care in Serbia is free at the point of use for registered refugees staying in state-run asylum or transit centres. Those living outside these camps are entitled to emergency care only. For pregnant women, mothers and infants, maternal and new-born health care is accessed through camp-based primary care doctors, who do not directly provide any level of care but refer to hospitals, with transportation being organised by the International Organisation for Migration (IOM).

Small-scale attempts to provide culturally appropriate care have been made by NGOs through the provision of cultural mediators, who accompanied women during hospital appointments, offering translation and advocating for the women, increasing acceptability and quality of care, as well as trust. In addition, the Serbian humanitarian organisation ADRA Serbia ran nurse-facilitated workshops for forcibly displaced women, translated by cultural mediators into Arabic and Farsi. The workshops covered various aspects of women’s health, including maternal and new-born health, that were tailored to the specific cultural context of attendees. Through creating a comfortable and safe space in which women could socialise and attend women’s health workshops while the children being looked after by ADRA staff, health literacy and knowledge regarding health access was provided. However, for the unstable funding of humanitarian assistance outside the regular health system and the dependence of individual commitment, the sustainability of this initiative is unclear.

**Thematic box**

**Culturally sensitive services in the humanitarian sector**

Long overlooked, cross-cultural considerations are increasingly discussed in relation to humanitarian assistance.^72^^,^^73^ Principle challenges lie in the tensions that may exist between the contextual adaptation of emergency care services as mentioned in the Sphere guidelines,^74^ and the possibility that international staff arriving in a humanitarian crisis setting with insufficient preparation regarding local realities offering culturally inappropriate care.^73^ The emergency frame, that is at the core of temporary humanitarian health structures, is likely to interfere with developing meaningful relations of care conducive to inclusive and safe experiences of health services. Research has shown, for example, that the framing of the so-called 2015 “migration” or “refugee crisis” has enabled a configuration of emergency care for newcomers in Greece and Italy, with reduced services and deterring well intentioned health professionals from reassessing pre-existing stereotypes or perceiving the agency of women when seeking medical care.^64^ In conjunction with “austerity-driven underfunding, temporary solutions became entrenched, producing a lasting emergency”.^64^^:11^

In all areas of humanitarian medicine, however, the lived experiences and positionality of health professionals who themselves experienced displacement and migration should be systematically involved. Both for training and humanitarian assistance, migrant professionals provide a renewed lens on cultural safety from the ground up.^75^

## Contributors

STS, MK, DD and KB conceived this article. STS and MK wrote the first draft, including sections written by DD, KB, ES and MIU-G. All authors contributed equally to reviewing and editing the manuscript and agree with the final version.

## Declaration of interests

We declare no competing interests.
